# Factors Affecting Phage D29 Infection: A Tool to Investigate Different Growth States of Mycobacteria

**DOI:** 10.1371/journal.pone.0106690

**Published:** 2014-09-03

**Authors:** Benjamin M. C. Swift, Zara E. Gerrard, Jonathan N. Huxley, Catherine E. D. Rees

**Affiliations:** 1 School of Biosciences, University of Nottingham, Sutton Bonington Campus, Loughborough, Leicestershire, United Kingdom; 2 School of Veterinary and Medicine Science, University of Nottingham, Sutton Bonington Campus, Loughborough, Leicestershire, United Kingdom; Rockefeller University, United States of America

## Abstract

Bacteriophages D29 and TM4 are able to infect a wide range of mycobacteria, including pathogenic and non-pathogenic species. Successful phage infection of both fast- and slow-growing mycobacteria can be rapidly detected using the phage amplification assay. Using this method, the effect of oxygen limitation during culture of mycobacteria on the success of phage infection was studied. Both D29 and TM4 were able to infect cultures of *M. smegmatis* and *Mycobacterium avium* subspecies *paratuberculosis* (MAP) grown in liquid with aeration. However when cultures were grown under oxygen limiting conditions, only TM4 could productively infect the cells. Cell attachment assays showed that D29 could bind to the cells surface but did not complete the lytic cycle. The ability of D29 to productively infect the cells was rapidly recovered (within 1 day) when the cultures were returned to an aerobic environment and this recovery required *de novo* RNA synthesis. These results indicated that under oxygen limiting conditions the cells are entering a growth state which inhibits phage D29 replication, and this change in host cell biology which can be detected by using both phage D29 and TM4 in the phage amplification assay.

## Introduction

MAP is an extremely slow-growing member of the *Mycobacterium* genus that can take up to 16 weeks to reach detectable levels using traditional culture methods [1]. It is known that pathogenic mycobacteria can persist and survive within macrophages when infecting their host and also that low levels of oxygen can induce dormancy in *M. smegmatis* [2] and *M. tuberculosis* [3]. It is speculated that MAP also has the ability to enter this dormant phase when grown under oxygen limiting conditions [4]. Physiological changes, such as cell wall thickening, have been reported in mycobacteria cells during dormancy [5] and in MAP changes in the expression of 55 proteins involved in hypoxia and starvation were identified [6] all of which may have a role in altering the ability of bacteriophage to infect their host mycobacterial cell.

Some mycobacteriophage, such as D29, TM4, L5 and Bxz2, have been isolated and are found to have a very broad host range so therefore must bind to receptors found on many different mycobacterial cell types [7]. While mycobacteriophage host infection is expected to be strongly determined by the availability of specific cellular receptors, few of these have been identified or studied [8], therefore little is known about the growth conditions needed to ensure that these receptors are expressed to promote good phage infection. In the past bacteriophage have proved to be important tools for interrogating the genetics and physiology of their pathogenic hosts [9]. In mycobacteria, they have been used to rapidly report on the antibiotic resistance of the host cell and rapid phage-based detection methods have been developed [10]. For instance phage amplification assays monitor the rapid growth of the phage which is faster than the growth rate of their mycobacterial host cell. The phage most commonly used to detect MAP is D29. For MAP, this assay has been used as rapid alternative to culture to detect and enumerate MAP in samples of blood, milk and cheese [10]–[13].

The aim of this investigation was to use the phage amplification assay as a tool to investigate how different growth and storage conditions of mycobacteria affect the host cell physiology, and thereby affect the efficiency of mycobacteriophage infection of the host cells.

## Materials and Methods

### Bacterial strains, bacteriophage and growth media

Two well described MAP cattle reference strains used were K10 and ATCC 19698 and one clinical cattle isolated kindly gifted by Dr Karen Stevenson; DVL 943.The *Mycobacterium smegmatis* strain used was mc^2^155, which is used routinely in phage assays [14]. All liquid cultures were grown in Media Plus (MP; Middlebrook 7H9/OADC [Becton Dickenson] supplemented with 2 mM CaCl_2_ [15]) or grown on Middlebrook 7H10/OADC agar slopes. For growth of MAP the media was supplemented with Mycobactin J (2 µg µl^−1^; Synbiotics Corporation, France). Bacteriophage D29 and TM4 were propagated with *M. smegmatis* on 7H10 agar [15].

### Detection, enumeration and antibiotic sensitivity testing of MAP

Detection, enumeration and antibiotic sensitivity testing was carried out according to [10]. Briefly, to perform the phage amplification assay, 1 ml samples containing mycobacteria were mixed with 1×10^8^ mycobacteriophage D29 (100 µl) and incubated for 1 h. After this time any remaining extracellular phage were inactivated using a virucide treatment (100 µl 10 mM ferrous ammonium sulphate) for 5 min. The virucide was then neutralised by dilution using 5 ml MP and the phage-infected cells were plated in a lawn of fast growing *M. smegmatis* (1 ml, 10^7^ CFU ml^−1^) using soft agar (0.8% w/v). Lysis of the infected cells at the end of the lytic cycle leads to the formation of a plaque in the lawn of *M. smegmatis*. Since each plaque formed represents one mycobacterial cell in the original sample the assay can be used to enumerate the mycobacterial cultures (data reported as PFU ml^−1^ [10]). Antibiotic sensitivity/MIC experiments were carried out by adding rifampicin (5 µg ml^−1^; RIF; Mast Diagnostics, UK). Phage will not replicate in RIF^S^ mycobacterial cells and no plaques will form at the end of the assay [10].

### Phage amplification assay using TM4

The virucide used for TM4 was made from green tea (Gunpowder tea, Whittards of Chelsea, UK) using the method described by [16]. Briefly, the tea was prepared by adding sufficient RO water to the tea solids (7% w/v) and the samples were boiled for 10 min. The infusion was then filtered (Whatman Grade No. 2 Filter Paper, Whatman International Ltd.), autoclaved and stored at 4°C. The phage amplification assay was performed as described above, apart from 100 µl of tea infusion was used as the virucide and the virucide incubation time increased to 15 min.

### Growth culture conditions

A method based on the Wayne model [3] was used to induce a non-replicating stationary phase in mycobacteria. Briefly the mycobacteria were grown in glass vials (12 ml volume) with a screw top lid filled to leave a head space ratio of 1∶2 (air: liquid) and the tops sealed to finger tight (for oxygen-limited growth). The samples were then incubated at 37°C whilst shaking at 250 rpm. For the oxygenated cultures, cells were grown at 37°C with shaking (250 rpm) in the same vials with the lids loosely closed.

### Phage attachment assay

The phage attachment assay was carried out as described by [17]. Briefly, the phage (approx. 10^7^ PFU ml^−1^) were added to each of the samples and incubated for up to 60 min to allow binding to their host cells. The samples were then centrifuged (13000×*g*; 3 min) to remove the MAP cells and any bound phage. Unbound phage remaining in the supernatant were then titred.

### Reversible inhibition of RNA synthesis using rifampicin

RIF-sensitive MAP cells, as determined by [18], were treated with an inhibitory concentration of RIF (5 µg ml^−1^) for 24 h. To remove the antibiotic, cells were recovered by centrifugation (13000×*g*; 3 min) and washed twice with MP, then resuspended in 1 ml of MP before further analysis performed.

## Results

### Detection of *M. smegmatis* cells using D29 and TM4

*M. smegmatis* was initially used as a fast-growing model organism for MAP. First *M. smegmatis* cells were grown to 1×10^7^ CFU ml^−1^ either aerobically or under conditions where oxygen would become self-limiting as growth of the culture occurred (Wayne's model [3]) which took 10 d in a 37°C incubator shaking at 200 rpm. Each day, samples (100 µl) were removed for viable count determination and for enumeration using the phage amplification assay.

**Figure 1:**
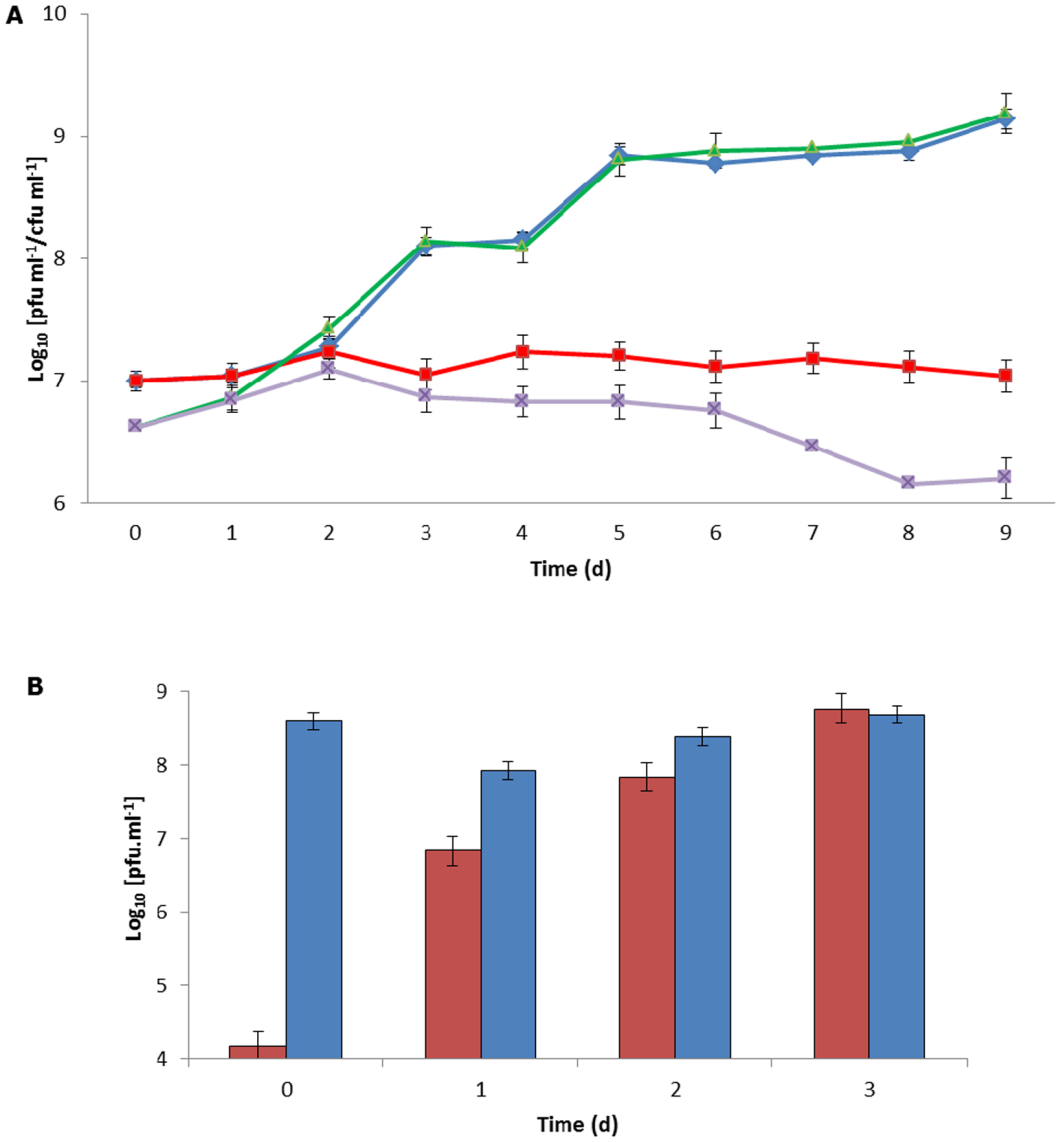
Comparison of *M. smegmatis* cells detected by phage and viable count under growth limiting and aerobic conditions. Panel A Graph showing the results of the phage assay (PFU ml^−1^; green and purple) and viable count (CFU ml^−1^; blue and red) for *M. smegmatis* cultured over a 10 d period under self-limiting oxygen conditions (red and purple) or under conditions were free oxygen exchange occurred (blue and green). T = 0 are values recorded for initial cultures before incubation. Error bars represent the standard deviations of the means of number of plaques recovered from the phage assay performed in triplicate. Panel B Graph showing the number of *M. smegmatis* cells detected using the phage amplification assay (PFU ml^−1^). Prior to dilution into fresh medium the *M. smegmatis* cells were either grown under self-limited oxygen conditions (red bars) or aerobic conditions (blue bars). Samples were taken from the fresh cultures over a 3 d period. Error bars represent the standard deviations of the means of number of plaques recovered from the phage assay performed in triplicate.

The results (Fig. 1A) show that there was no difference (P>0.05) in the number of *M. smegmatis* cells detected by culture (CFU ml^−1^) or by the phage assay (PFU ml^−1^) when they were cultured aerobically over the whole time series, demonstrating that the phage amplification enumeration assay compared well with traditional culture when used on aerobically grown *M. smegmatis* cells. However when the *M. smegmatis* was grown in oxygen limiting conditions, the PFU ml^−1^ values were almost one log_10_ lower than the CFU ml^−1^ value recorded after 10 d (Fig. 1A) showing that the phage were no longer able to detect the *M. smegmatis* cells efficiently when they were cultured under these conditions.

To determine whether the undetectable nature of the cells could be reversed, the *M. smegmatis* cells grown under oxygen limited conditions were diluted into fresh MP and incubated aerobically (shaking at 37°C at 200 rpm) and tested for 3 d and this was compared against the aerobically grown cells. At time point 0 approximately 10^4^ PFU ml^−1^ of *M. smegmatis* cells were detected. After 1 d of aerobic incubation, the phage assay was able to detect nearly 10^7^ PFU ml^−1^. The number of cells detected in the culture transferred from the anaerobic growth conditions increased until after 3 d there was no difference (P>0.05) in PFU ml^−1^ values obtained for both cultures (Fig. 1B).

TM4 is broad spectrum mycobacteriophage that has been postulated to have the ability to infect non-replicating mycobacteria in the stationary phase of growth. Since the virucide used for D29 does not inactivate TM4 efficiently, tea infusions were tested for use as a virucide in the phage amplification assay as previously described [16]. The tea extract was shown to cause a 6-log_10_ destruction of TM4 within 15 min while having no adverse effect on the viability of mycobacteria tested (both MAP and *M. smegmatis*; Fig. S1). Using these extracts the *M. smegmatis* experiment was repeated using phage TM4 as well as phage D29.

**Figure 2:**
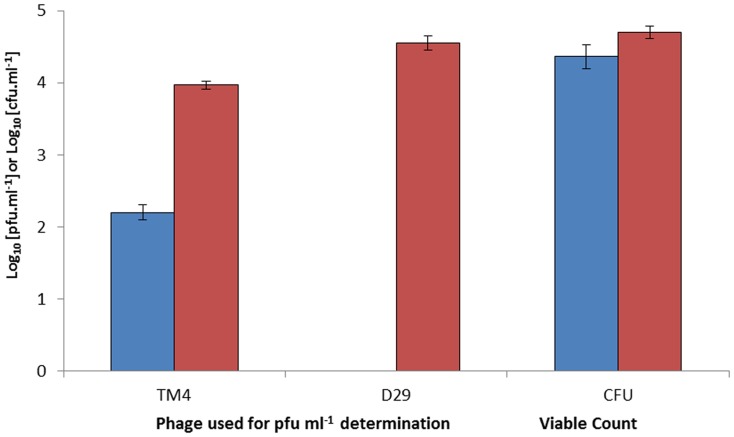
Detection of *M. smegmatis* using D29 and TM4. Graph showing the number of plaques recovered following the phage assay when using phage TM4 and D29. The *M. smegmatis* cells tested were either grown with limiting oxygen (blue bars) or after these cells had been grown with aeration (red bars). In addition to the phage assay the viable count (CFU ml^−1^) of both cultures was determined. Error bars represent the standard deviations of the means of number of plaques and colonies recovered from the phage assay and viable count, respectively, performed in triplicate.

The viable count of the *M. smegmatis* cultures grown aerobically or under oxygen limiting conditions were very similar (both approx. 1×10^4^ CFU ml^−1^; Fig 2). The results of the phage amplification assays showed that TM4 was able to infect *M. smegmatis* grown under both conditions, although the efficiency with which cells were detected was lower (only 1.58×10^2^ PFU ml^−1^ detected in both samples; Fig. 2). In contrast D29 was not able to infect the cells grown under limiting oxygen conditions at all but for the cells growing in the presence of oxygen, the number of cells detected by D29 was not significantly different (P>0.05) from the viable count (1.6×10^4^ CFU ml^−1^). These results indicate that D29 is more efficient at infecting *M. smegmatis* cells grown under aerobic conditions than TM4 in this medium.

### Detection of MAP cells using D29 and TM4

To determine whether the growth conditions affected the ability of phage D29 to infect a slow-growing mycobacterial species, this experiment was repeated using three strains of MAP (K10, DVL 453 and ATCC 19851). When cultures were grown using the Wayne's model conditions for 1 month, no MAP cells detected using phage D29 although growth was visible in the cultures. Cells from this phage-undetectable culture were then inoculated into fresh MP (100 µl cells into 9.9 ml MP) without Mycobactin-J. This was to limit growth so that recovery of cells from the uninfectable state would be more apparent.

**Figure 3:**
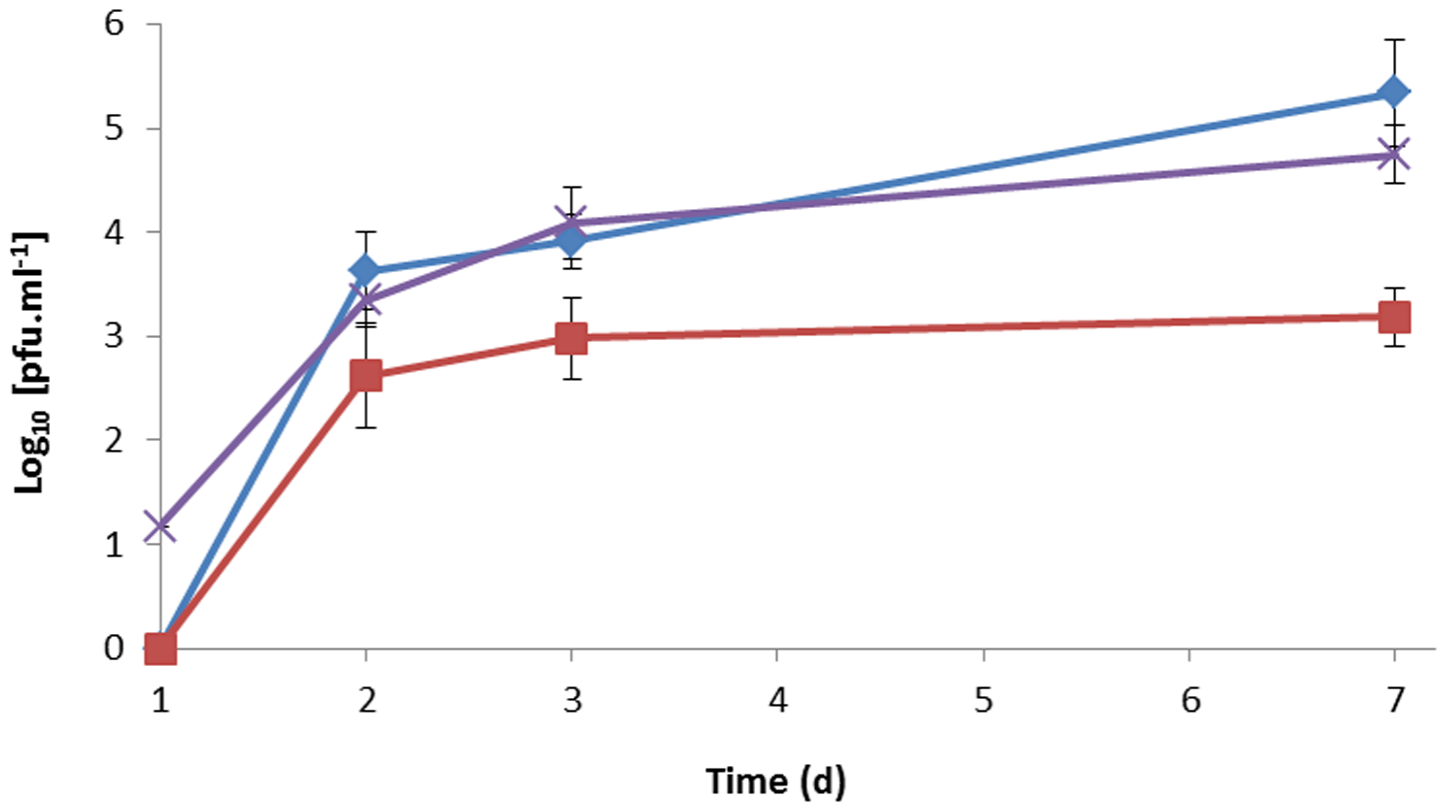
Recovery of phage D29 infectivity by three strains of MAP. Graph showing the number of MAP cells detected using the RapidMAP assay (PFU ml^−1^). Prior to dilution into fresh medium the MAP cells were either grown under self-limiting oxygen conditions and then samples were taken from the fresh cultures over a 7 d period. The three strains of MAP used were K10 (blue), DVL 453 (purple) and ATCC 19851 (red). Error bars represent the standard deviations of the means of number of plaques recovered from the phage assay performed in triplicate.

After one day incubation with aeration, only cells in the DVL 453 strain were detectable using the phage amplification assay (1.5×10^1^ PFU ml^−1^; Fig. 3). On day 2, a 2–3 log_10_ increase in the number of cells detected by the phage assay was seen for each strain of MAP tested. After 7 days of aerated incubation, the number of MAP cells detected using D29 in the phage amplification assay was between 10^2^ to 10^5^ PFU ml^−1^. As there was no Mycobactin-J added to the cultures this suggest that there was an increase in infectivity rather than an increase in cell number.

**Figure 4:**
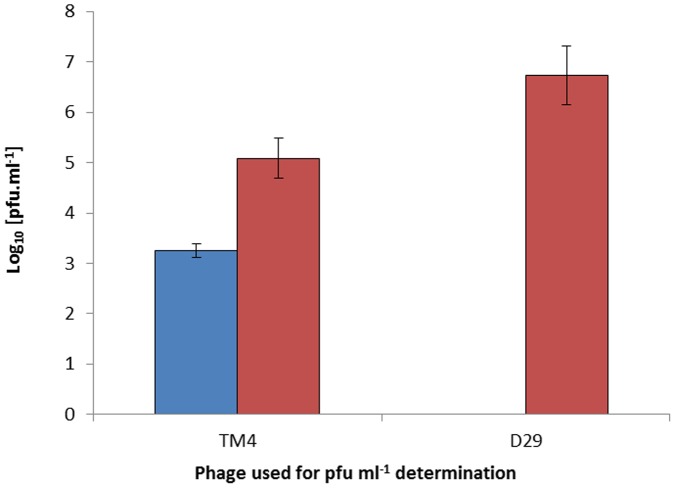
Difference in infectivity of MAP cells by D29 and TM4. Graph showing the number of plaques recovered following the phage assay when using phage TM4 and D29. The MAP cells tested were either grown with limiting oxygen (blue bars) or after these cells had been exposure to air for 9 days (red bars). Error bars represent the standard deviations of the means of number of plaques recovered from the phage assay performed in triplicate.

When the experiment was repeated with phage TM4 the results show that for cells grown under oxygen limiting conditions 1.8×10^3^ PFU ml^−1^ MAP were detected whereas, once again, D29 did not detect any MAP cells (Fig. 4). As before, when these cells were exposed to air for 9 d, phage D29 was able to detect significantly more MAP cells (1.5 log_10_ PFU ml^−1^; P<0.01) compared to TM4, confirming that phage D29 is able to infect the mycobacteria more efficiently than TM4 when the cells are well aerated in this media.

### Attachment of phage D29 to non-detectable MAP cells

**Figure 5:**
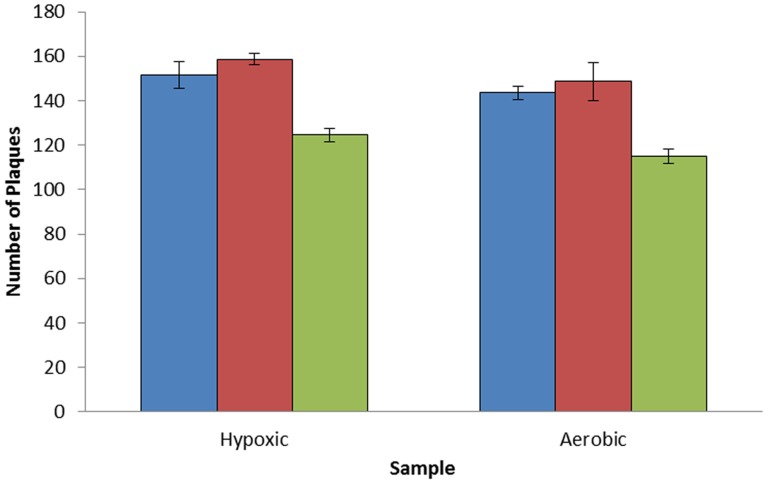
Effect of stationary phase bacteria on the attachment of phage D29 to MAP cells. Graph showing the number of unbound phage particles to MAP cells that are infectable (aerobic) and uninfectable (hypoxic) after 0 min (blue bars), 30 min (red bars) and 60 min (green bars). Error bars represent the standard deviations of the means of number of bacteriophage detected after each time point in triplicate.

To investigate what might be preventing successful D29 phage infection, a phage attachment assay was performed. MAP cells (1×10^5^ PFU ml^−1^) were grown for 1 month under oxygen limiting conditions (using Wayne's model) and aerobically. The samples were then mixed with phage D29 (1×10^7^ PFU ml^−1^) for 0, 30 and 60 min to allow attachment of phage to the cells. Infected cells/phage attached to cells were then removed from the culture by centrifugation and the number of phage remaining in the supernatant titred. The results show that there was an approximate 20% drop in the number of free phage particles present in the culture supernatant for both cell cultures after 60 min of incubation with the phage (Fig. 5), and there was no statistical difference (P>0.05) in the level of attachment of phage to the cells in the two cultures. When bacteriophage were added to media without cells, there was no reduction in phage titre, showing that the loss of phage from the supernatant was due to the attachment of phage to the cells. This result suggests that the D29 receptors are not altered or lost when the mycobacteria are grown under oxygen limiting conditions, but productive infection is blocked.

### Role of RNA synthesis inhibition on phage infection

Antibiotics that block RNA synthesis can be used to determine if *de novo* protein synthesis is required for an adaptive event to occur. However, before determining whether differences during dormancy were the reason for the inhibition of phage infection, the effect of the antibiotic on resuscitation of hosts was investigated. First the sensitivity of the MAP K10 cells to RIF was determined using the *FASTPlaque*TB™ Response protocol [10]. To do this MAP K10 cells were treated with an inhibitory concentration of RIF (5 µg ml−1) and the phage assay was then performed. The results showed that the MAP cells were sensitive to RIF since plaque formation was inhibited completely. To then determine whether the RIF inhibition of RNA polymerase was reversible for MAP, the antibiotic was removed from RIF-treated cells by centrifugation (13000×g; 3 min) and washing twice with MP. In this case plaques were formed showing that productive phage replication is RIF sensitive and that RIF inhibition is reversible and the number of plaques formed were not significantly different (P>0.05) to the numbers before RIF treatment (Fig. S2).

**Figure 6:**
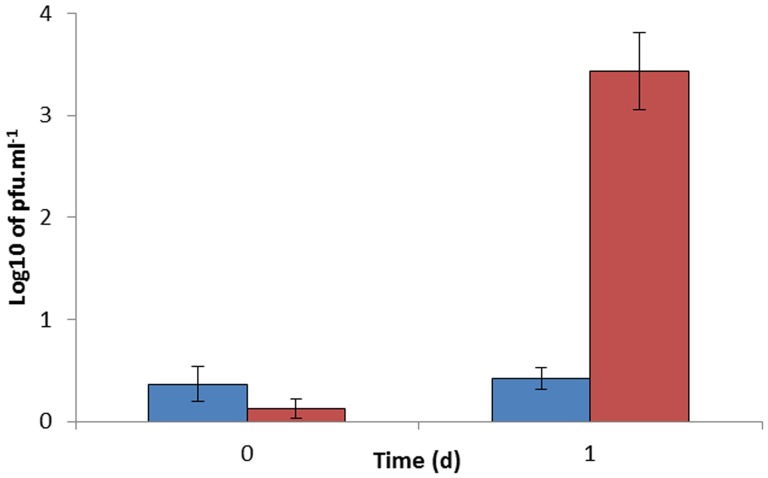
Role of RNA synthesis in recovery of phage-sensitivity. Graph showing the number of MAP cells detected by the RapidMAP assay, after uninfectable MAP cells were treated with RIF (blue bars) and without RIF (red bars) before exposure to oxygen (t – 0) and after exposure to oxygen (t – 1). Error bars represent the standard deviations of the means of number of plaques recovered from the phage assay performed in triplicate.

To determine whether D29 phage infection was inhibited by *de novo* protein synthesis, antibiotics were used to transiently inhibit RNA synthesis in the host mycobacteria. MAP cells were grown for 1 month under oxygen-limiting conditions to induce the uninfectable state, and then treated with RIF. After one day aeration, there was a significant (P<0.01) three-log_10_ increase in the number of MAP cells detected in the sample that was not treated with RIF (Fig. 6). However after the antibiotic was washed out of the RIF-treated cells using the method described above, no MAP cells were detected in the RIF-treated sample. This result suggests that there is a requirement for *de novo* gene expression for conversion of the MAP cells back into and infectable state.

## Discussion

The phage amplification assay is a powerful tool that can detect, enumerate and determine the antibiotic sensitivity of mycobacteria. However, a lack of information about phage-host interactions may lead to inaccurate interpretation of results. Phage D29 was found to be unable to infect both MAP and *M. smegmatis* cells when they were induced into a non-growing phase, when oxygen became self-limiting in the growth tubes, as described by [3]. However, when the cells were reintroduced to oxygen, the ability of the phage to infect the cells was restored almost completely although the time required to achieve this was different for the two organisms tested; three days for *M. smegmatis* and over one week for MAP. This result probably reflects the very different natural growth rates of these two bacterial species.

*M. smegmatis, M. bovis* and *M. tuberculosis* have all been reported to have the ability to enter a ‘non-replicating’ stationary phase during hypoxic stress [2], [3], [19]. Hypoxia is predicted to be a key host-induced stress, limiting growth of pathogenic mycobacteria *in vivo*. Many studies have indicated that *M. tuberculosis* adapts to oxygen limitation by entering into a metabolically altered state while awaiting the opportunity to reactivate [20]. Interestingly, using D29 to perform the phage amplification assay, we have been able to sensitively detect MAP in white blood cells isolated from the blood of naturally infected animals [9]. Our discovery concerning the effect of hypoxia on the efficiency of D29 infection suggests that these cells recovered from clinical samples are not in a state of hypoxic stress and perhaps then are not dormant. Whittington *et al*. [4] presented evidence that MAP present on grass could enter a dormant state similar to that described for *M. tuberculosis* and suggested that this may facilitate the survival of this organism in the environment, however when present on the surface of grass it would not be anticipated that oxygen was limiting for these cells.

It is known that adsorption of D29 to *M. tuberculosis* is more efficient when the bacteria are in exponential phase of growth [21]. The reduction in the adsorption efficiency was thought to be due to structural changes on the cell wall of the host, such as cell wall thickening due to the accumulation of alpha-crystallin chaperone protein [3], [5], which occur when they are not in the exponential growth phase, and this in turn may affect the accessibility of D29 to specific phage receptor sites [21]. Recently some MAP strains have been reported to undergo morphological changes during nutrient starvation [22] and cell wall thickening could prevent phage D29 from attaching to the cell surface and binding its receptor. However in this study the phage attachment assay suggests there was no change in the ability of the phage to bind to MAP cells, and therefore there this suggests that simple changes in the cell wall structure alone do not explain the inhibition of productive phage infection.

When MAP cells induced into the hypoxic non-infectable state were treated with RIF to prevent *de novo* protein synthesis, the cells were unable to fully revert to an infectable state until the RIF was washed out of the cells, suggesting that there is a requirement of RNA synthesis and *de novo* gene expression for the transition of the cells to active aerobic growth. Researchers have found that a homologue of the DNA binding-like protein (Dps) in the *M. avium* genome, which was first identified in *M. smegmati*s and confers protection by binding to DNA during nutritional and oxidative stress in other bacteria [4]. Dps has been shown to confer resistance to bacteriophage that infect *E. coli*. Dps was found to be present in phage sensitive *E. coli* cells and was thought to be the reason for the phage resistance [23]. Thus resistance to D29 phage infection may be due to an accumulation of proteins such as Dps which would bind to the replicating phage DNA and prevent productive phage replication and it may require the expression of proteases or other regulatory proteins to remove the accumulated Dps from the cells.

In contrast to D29, bacteriophage TM4 has been reported to be able to infect stationary phase mycobacteria [24], [25]. It has been postulated that this difference is due to a peptidoglycan hydrolase motif found on the tape measure protein of TM4 that is not present on the tail of D29. This motif is thought to act in a similar way to resuscitation protein factors (Rpfs) which can induce stationary phase mycobacteria cells into an active growth state [26] by generating a signal that mimics the Rpfs leading to mycobacteria resuscitation, and therefore productive TM4 replication. It was also notable that TM4 infection of both *M. smegmatis* and MAP was not as efficient as that achieved by phage D29 when aerated cultures were used. However this may simply reflect the fact that the growth media used for the phage amplification assay was optimised for D29 infection and contains 2 mM CaCl_2_, which has been reported to inhibit TM4 infection of *M. smegmatis* and *M. bovis* BCG cells [27].

The results in this study demonstrate that bacteriophage TM4 and D29 can be used as a tool to identify when MAP adapts to hypoxic stress. As tools, they could be used to determine how many other stress conditions, such as UV and pH [4] also induce this response and whether different environmental conditions induce difference responses in the host that can be detected by changes in efficiency of phage infection. It would be interesting to identify genes required to be expressed to allow transition to occur as way of understanding the basis of this adaptive response and these phage would provide a good tool to screen for such genes.

## Supporting Information

Figure S1 **The ability of tea and FAS to inactivate phage TM4 and D29.** Graph showing the number of phage particles remaining after treatment with green tea (Tea) and ferrous ammonium sulphate (FAS). Error bars represent the standard deviations of the mean number plaques recovered from the phage titre.
(TIF)

Figure S2 **The effect of washing out RIF on MAP cell detectability with the phage assay.** Graph showing whether phage D29 can infect RIF^s^ MAP cells exposed to RIF and when RIF is washed away. Error bars represent the standard deviations of the mean number plaques recovered from the phage amplification assay.
(TIF)
