# Supplementary figures and images for: Factors Affecting Phage D29 Infection: A Tool to Investigate Different Growth States of Mycobacteria
Source: PLoS One. 2014 Sep 3;9(9):e106690. doi: 10.1371/journal.pone.0106690 (PMC4153674; doi:10.1371/journal.pone.0106690)

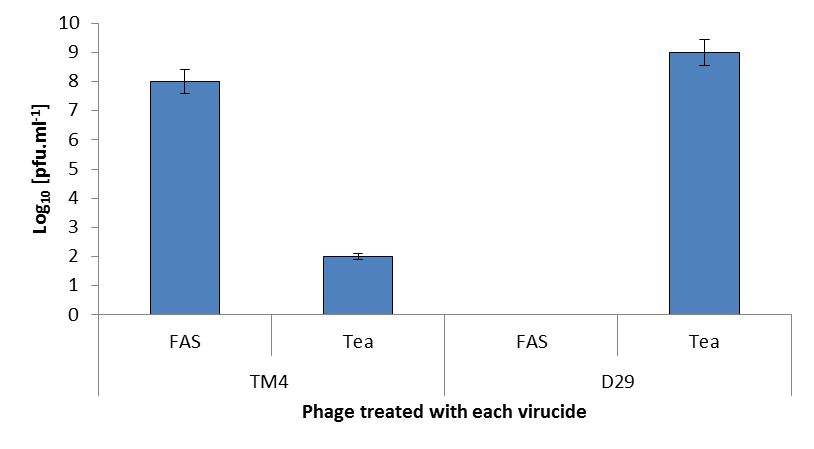

Supplement: Figure S1 — The ability of tea and FAS to inactivate phage TM4 and D29. Graph showing the number of phage particles remaining after treatment with green tea (Tea) and ferrous ammonium sulphate (FAS). Error bars represent the standard deviations of the mean number plaques recovered from the phage titre. (TIF) [file pone.0106690.s001.tif]

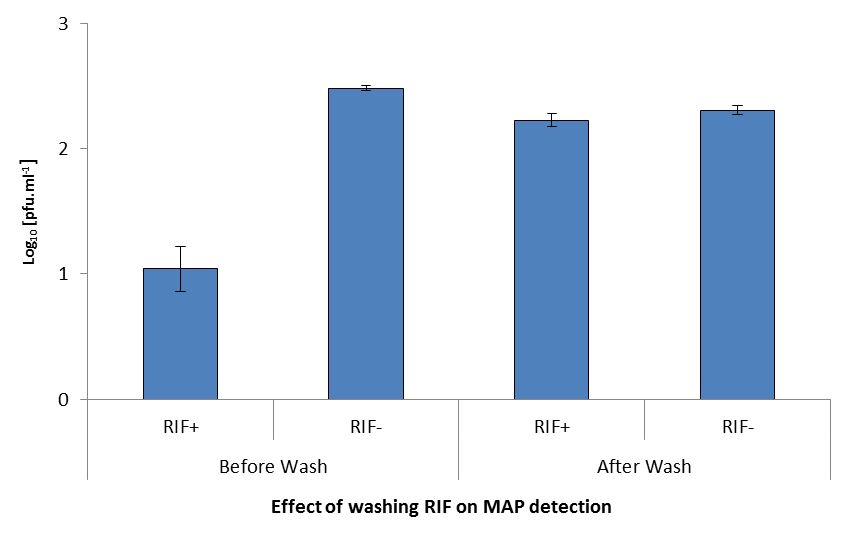

Supplement: Figure S2 — The effect of washing out RIF on MAP cell detectability with the phage assay. Graph showing whether phage D29 can infect RIFs MAP cells exposed to RIF and when RIF is washed away. Error bars represent the standard deviations of the mean number plaques recovered from the phage amplification assay. (TIF) [file pone.0106690.s002.tif]
